# Preparation of three-dimensional palygorskite based carrier

**DOI:** 10.1016/j.mex.2020.100815

**Published:** 2020-02-20

**Authors:** Yi Wang, Yuxia Shen, Ziyi Qin, Shuang Li, Ting Zhang

**Affiliations:** College of Petrochemical Technology, Lanzhou University of Technology, Lanzhou 730050, China

**Keywords:** Functionalized palygorskite, Diisocyanate, Porous carrier, Grafting from, Grafting onto

## Abstract

Palygorskite is a kind of crystalline hydrated magnesium aluminum silicate mineral with micro-fibrous morphology. Due to the large specific surface area, moderate cationic exchange capacity and pronounced adsorption properties, it has been widely used in many fields. In order to enhance the loading capacity and adjust the microstructure of palygorskite (Pal) crystal, series of three-dimensional palygorskite carriers (3D Pal) with different pore size were fabricated through grafting from or grafting onto method. Due to the functional and cross-linked molecules act as upholder reagents, the specific surface of individual palygorskite is fully utilized and the load capacity is greatly improved. The porosity and pore size of 3D palygorskite based carrier also can be regulated by the length of organic molecular chain segments. The successful preparation of 3D Pal-based carrier provides a new way for surface grafting, modification or preparing 3D carrier of palygorskite and other minerals.

• The developed method allows fabricating three-dimensional palygorskite based carriers by covalent bonding method.
• The pore size of the as-prepared carriers can be conveniently adjusted by length of the bonded molecular chain.

## Graphical abstract

**Figure:**
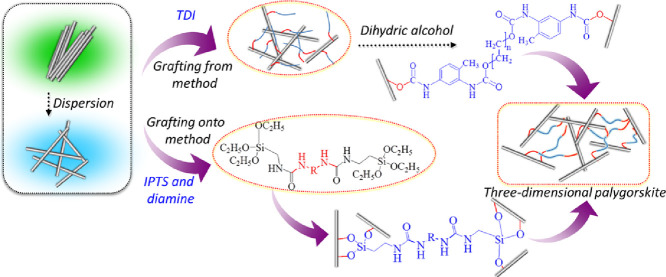

**Table:** Specification Table

Subject area:	Materials Science
More specific subject area:	Palygorskite modification; Three-dimensional palygorskite
Method name:	Surface grafting and covalent bonding
Name and reference of original method:	N/A
Resource availability:	N/A

## Method details

### Background

The current studies only focused on the utilization of natural and inherent characteristics of mineral materials. Although acid treatment, thermal activation and surface functionalization methods are employed to optimize the structure and loading capacity of mineral carriers [4,5], the locally improved microstructure and partially modified surface cannot change the characteristics fundamentally. The specific surface area of mineral units cannot be fully utilized because of the large free energy of nano-particles and their porous structures cannot be regulated. On the other hand, the morphology, crystallographic structure, microstructure (including strut, pore or cell sizes, specific surface area and particle size) and surface groups of the mineral carrier affect the properties of mineral-based composites [1], [2], [3]. In order to enhance the loading capacity of palygorskite (Pal), surface grafting and covalent bonding methods were adopted to fabricate Pal-based three-dimensional network, which took advantage of the active reactions between functional groups to graft organic molecules onto the surface of Pal, thereby achieving the aim of preventing the agglomeration of Pal crystals. Moreover, the porosity and pore size of three-dimensional Pal can be regulated by adjusting the length of organic molecular chain segments. Properly speaking, this study provides a new way for surface grafting, modification or preparing 3D carrier of palygorskite and other minerals. According to reaction mechanism, the methods can be divided into two different types, one is grafting from the surface, and the other is grafting onto the surface.

### Method description

**Fig. 1:**
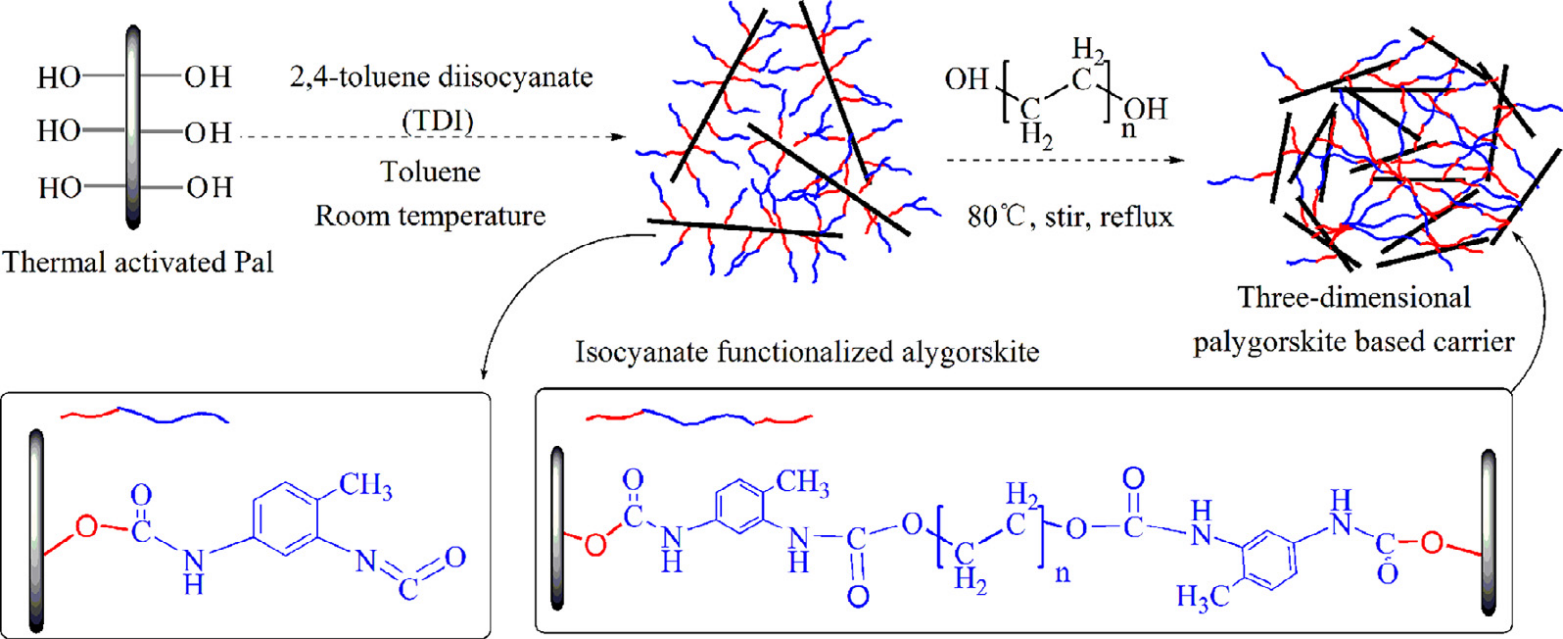
Synthesis mechanism diagram of 3D Pal using polyethylene glycol as bonding reagent.

**Fig. 2:**
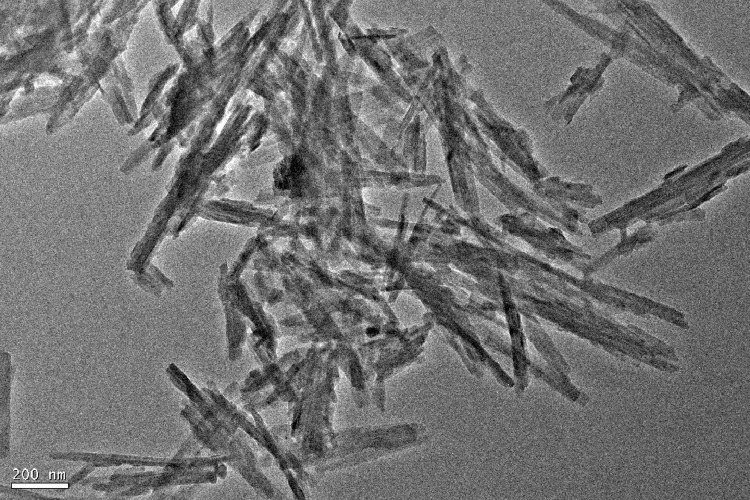
TEM image of 3D Pal synthesized with PEG6000.

**Fig. 3:**
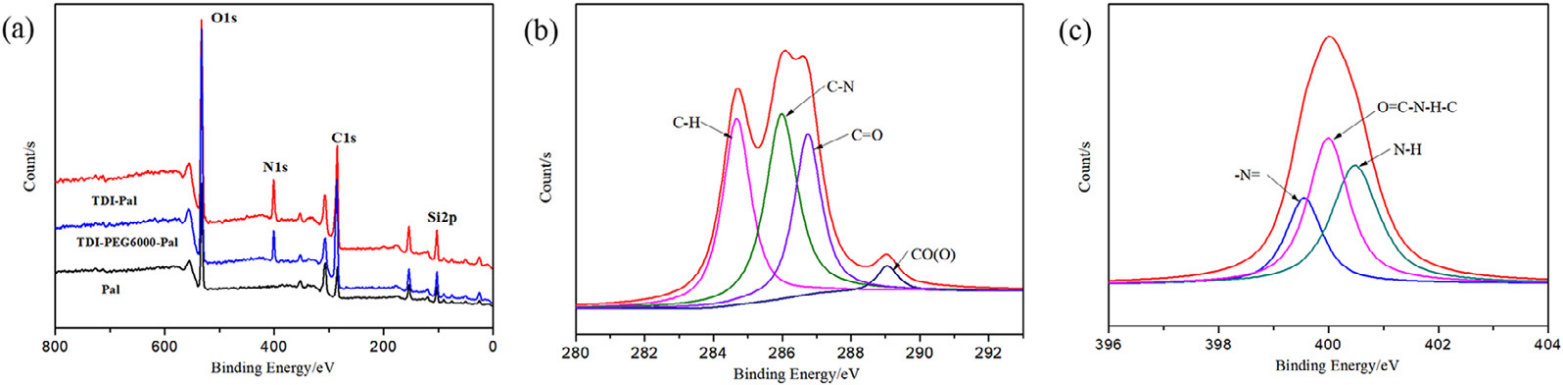
The full spectra (a) the C spectra (b) and the N spectra (c) of 3D Pal synthesized with PEG6000.

As for grafting from the surface of Pal, the specific technical means and experimental procedures are as follows. In the pretreatment process of Pal, water washing and acid treatment are included, and the pH of Pal solution needs to be adjusted to neutral before centrifugation. Before participating in the reaction, the Pal was dried at 105 °C for 5 h. The hydrochloric acid activated Pal or thermal treated Pal was added to a three-necked flask containing toluene solution and ultrasonic dispersed for 1 h Accordingly, toluene solution containing 2, 4-toluene diisocyanate (TDI) was introduced and grafted at room temperature for 24 h. In this step, Pal is functionalized with isocyanate groups (-NCO). Then, diols with different molecular weights and molar ratio of 0.25 (to TDI) were added to the above solution severally when temperature rose to 80 °C. At this time, the single Pal crystal will be covalent bonded by the reaction of remaining isocyanate groups on the surface of Pal with the hydroxyl groups of diols. After 8 h, three-dimensional palygorskite was obtained by washing successively with ethanol and deionized water, and freeze drying. In this procedure, the length of dihydric alcohol chain can be selected to obtain Pal based three-dimensional carrier with different pore sizes. Also, diol molecules can be replaced by other bifunctional compounds containing active hydrogen atoms, such as glycol, diamine and diacid. The typical preparation mechanism is shown in Fig. 1. The TEM image of 3D Pal synthesized with PEG6000 was displayed in Fig. 2 and the XPS spectra were shown in Fig. 3.

**Fig. 4:**
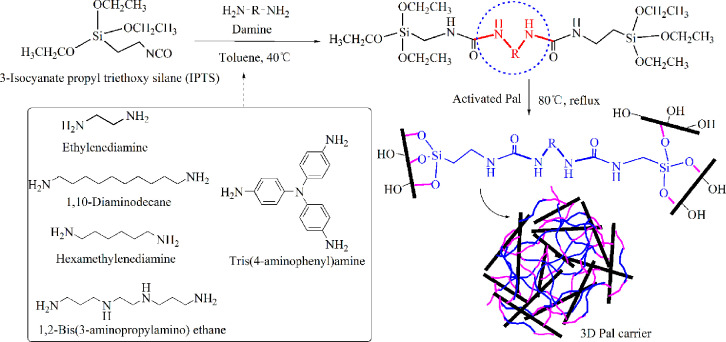
Synthesis mechanism diagram of 3D Pal using grafting onto method.

**Fig. 5:**
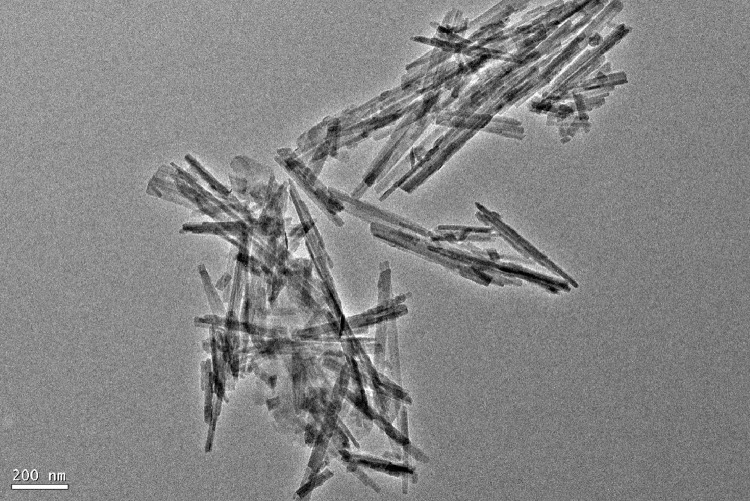
TEM image of 3D Pal synthesized with ethylenediamine.

**Fig. 6:**
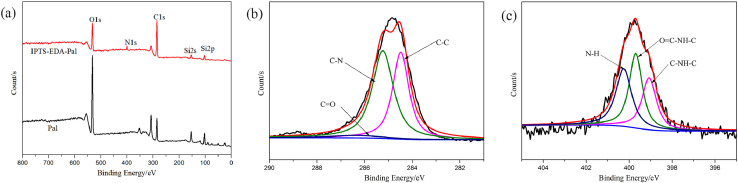
The full spectra (a) the C spectra (b) and the N spectra (c) of 3D Pal synthesized with ethylenediamine.

As for the grafting onto method, the technical steps and experimental procedures are as follows. According to amine group to isocyanate group mole ratio of 1:1, 4 mL 3-isocyanate propyl triethoxy silane (IPTS) and the corresponding amount of diamines were added into a three-necked flask containing 40 mL of toluene. Then, the mixed solution was stirred and refluxed for 2 h at 40 °C. At the same time, 10 g hydrochloric acid activated Pal or thermal treated Pal was added into a beaker containing 60 mL toluene and dispersed ultrasonically for 1 h Afterwards, the dispersive Pal slurry was put into the above three-necked flask and temperature raised to 80 °C. After 12 h reaction under reflux condition, the products were washed repeatedly with deionized water and extracted with absolute ethanol. Finally, loose powder was obtained after drying in a freeze dryer for 24 h. Similarly, different diamines can be selected, such as ethylenediamine, 1,6-hexamethylenediamine, 1,10-diaminodecane, 1,2-bis(3-aminopropylamino) ethane, tris (4-aminophenyl) amine, and etc. The mechanism diagram for preparation of 3D Pal using grafting onto method was demonstrated in Fig. 4. The TEM image of 3D Pal synthesized with ethylenediamine was displayed in Fig. 5 and the XPS spectra were shown in Fig. 6.

## Appendix Supplementary materials

Image, application 1
