# Supplementary material for: Preparation of three-dimensional palygorskite based carrier
Source: MethodsX. 2020 Feb 20;7:100815. doi: 10.1016/j.mex.2020.100815 (PMC7078386; doi:10.1016/j.mex.2020.100815)
Supplement: Supplementary file 1 [file mmc1.doc]

# Author Contribution Statement

# *Yi Wang: Conceptualization, Methodology, Writing - Review & Editing, Funding acquisition. Yuxia Shen: Writing - Original Draft, Visualization. Ziyi Qin: Writing - Original Draft, Investigation. Shuang Li: Investigation. Ting Zhang: Supervision, Project administration.*
